# A unified framework for managing provenance information in translational research

**DOI:** 10.1186/1471-2105-12-461

**Published:** 2011-11-29

**Authors:** Satya S Sahoo, Vinh Nguyen, Olivier Bodenreider, Priti Parikh, Todd Minning, Amit P Sheth

**Affiliations:** 1 The Kno.e.sis Center, Department of Computer Science and Engineering, Wright State University, Dayton, OH USA; 2 Center for Tropical and Emerging Global Diseases, Univeristy of Georgia, Athens, GA USA; 3 Lister Hill National Center for Biomedical Communications, National Library of Medicine, NIH, Bethesda, MD, USA; 4 Division of Medical Informatics, School of Medicine, Case Western Reserve University, Cleveland, OH USA

## Abstract

**Background:**

A critical aspect of the NIH *Translational Research *roadmap, which seeks to accelerate the delivery of "bench-side" discoveries to patient's "bedside," is the management of the *provenance *metadata that keeps track of the origin and history of data resources as they traverse the path from the bench to the bedside and back. A comprehensive provenance framework is essential for researchers to verify the quality of data, reproduce scientific results published in peer-reviewed literature, validate scientific process, and associate trust value with data and results. Traditional approaches to provenance management have focused on only partial sections of the translational research life cycle and they do not incorporate "domain semantics", which is essential to support domain-specific querying and analysis by scientists.

**Results:**

We identify a common set of challenges in managing provenance information across the *pre-publication *and *post-publication *phases of data in the translational research lifecycle. We define the semantic provenance framework (SPF), underpinned by the Provenir upper-level provenance ontology, to address these challenges in the four stages of provenance metadata:

(a) Provenance **collection **- during data generation

(b) Provenance **representation **- to support interoperability, reasoning, and incorporate domain semantics

(c) Provenance **storage **and **propagation **- to allow efficient storage and seamless propagation of provenance as the data is transferred across applications

(d) Provenance **query **- to support queries with increasing complexity over large data size and also support knowledge discovery applications

We apply the SPF to two exemplar translational research projects, namely the Semantic Problem Solving Environment for *Trypanosoma cruzi *(*T.cruzi *SPSE) and the Biomedical Knowledge Repository (BKR) project, to demonstrate its effectiveness.

**Conclusions:**

The SPF provides a unified framework to effectively manage provenance of translational research data during pre and post-publication phases. This framework is underpinned by an upper-level provenance ontology called Provenir that is extended to create domain-specific provenance ontologies to facilitate provenance interoperability, seamless propagation of provenance, automated querying, and analysis.

## Background

**Figure 1:**
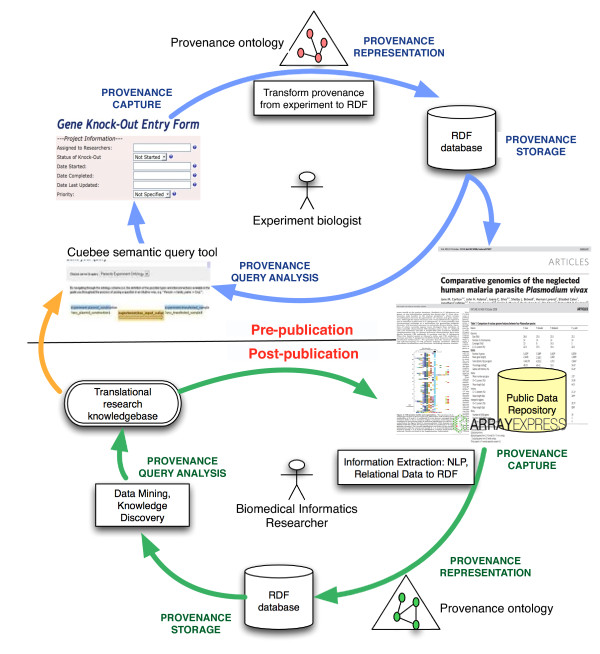
**Provenance lifecycle in the pre and post-publications stage of the translational research**. In the pre-publication phase, the biologist uses the provenance information, generated during an experiment, for project management and for publication in literature. In the post-publication phase, provenance, of both the process of information extraction and the extracted information, is used for ranking results and refining quality of results in data mining and knowledge discovery applications.

The key notion of translational research is the flow of information resources (experiment data, publications/literature, clinical trial data, or patient records) across organizations, domains, and projects that impacts both patient care and (through a feedback process) basic research. This necessitates keeping track of the provenance metadata of resources from the point of their creation to intermediate processing, and finally their end use. Provenance, derived from the French term *provenir *meaning "to come from", has traditionally played an important role in keeping track of cultural artifacts, such as paintings and sculpture, but is also rapidly becoming a key component of the high-throughput data generation and computing infrastructure used in translational research. Figure 1 illustrates the four phases of the provenance life cycle, both before the publication of scientific data and results in literature or submission to data repositories (*pre-publication*) and the use of the results by data mining or knowledge discovery applications (*post-publication*).

During the pre-publication phases (Figure 1), provenance is collected to describe the experiment design, such as details about the biological or technical replication (RNA extracts or cDNA clones) in microarray experiments, the type of parasite used to create an avirulent strain, or the demographic information used in a clinical trial [1]. Similarly, provenance information about the experiment platform (e.g. type of instruments used) and the tools used to process or analyze data (algorithms, statistical software) is also collected [2].

In the post-publication phase (Figure 1), data mining and knowledge discovery applications use provenance associated with the data extracted from peer-reviewed literature (e.g. PubMed), public data repositories (e.g. Entrez Gene), and Web resources (e.g. the European Bioinformatics Institute Web services) to guide analysis algorithms and interpretation of results [3]. Specifically, the provenance information in post-publication phase is used to constrain extraction processes to reputable sources (e.g. journals with a high impact factor), clustering datasets according to their source, and ranking results based on the timestamp or authorship information [3].

Figure 1 illustrates that the provenance metadata follows similar lifecycle phases in both pre- and post-publication stages, but each stage has distinct requirements. We introduce two exemplar translational research projects, each corresponding to a specific stage, to describe the challenges that need to be addressed for creating an effective provenance management system.

### The Semantic Problem Solving Environment for *T.cruzi *project (*pre-publication*)

*T.cruzi *is the principal causative agent of the human Chagas disease and affects approximately 18 million people, predominantly in Latin America. About 40 percent of these affected persons are predicted to eventually suffer from Chagas disease, which is the leading cause of heart disease and sudden death in middle-aged adults in the region. Research in *T.cruzi *has reached a critical juncture with the publication of its genome in 2005 [4] and can potentially improve human health significantly. But, mirroring the challenges in other translational research projects, current efforts to identify vaccine candidates in *T.cruzi *and development of diagnostic techniques for identification of best antigens, depend on analysis of vast amounts of information from diverse sources. To address this challenge, the Semantic Problem Solving Environment (SPSE) for *T.cruzi *project has created an ontology-driven integration environment for multi-modal local and public data along with the provenance metadata to answer biological queries at multiple levels of granularity [5].

**Figure 2:**
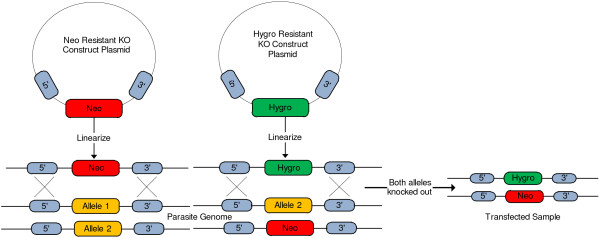
**Schematic representation of the procedure to knock out both the alleles of a gene during the transfection process**. The alleles for a particular gene are knocked out to totally ablate (or atleast reduce) the function of the gene and are replaced with the selected antibiotic (neomycin or hygromycin) resistance gene during the transfection experiment process.

Reverse genetics is one of the several experiment methods used in the study of the *T.cruzi *parasite and involves the creation of avirulent (non-virulent) strains of the parasite in the laboratory [6]. The process to create a new strain (Figure 2) may take many months involving multiple researchers or experiment techniques, and at each step, provenance information must be collected and stored to allow researchers and administrators to track and manage the experiments. The relevant provenance information includes, samples identifier, names and annotation information for the targeted genes, justification for knockout, plasmid constructs, antibiotic resistance genes, transfection methods (e.g. sonication, electroporation), number of transfection attempts, selection antibiotic, period of selection, and the ultimate success of knocking-out the gene from the genome.

Traditionally, bench science has used manual techniques or ad-hoc software tools to collect and store provenance information (discussed further in the Discussion and Related Work section). This approach has several drawbacks, including the difficulty in ensuring adequate collection of provenance, creation of "silos" due to limited or no support for provenance interoperability across projects. Further, the use of high-throughput data generation technologies, such as sequencing, microarrays, mass spectrometry (ms), and nuclear magnetic resonance (NMR) are introducing additional challenges for the traditional approaches to provenance management. A new approach for provenance management is also required to support the increasing trend of publishing experiment results (e.g. microarray data) to community data repositories (e.g. European Bioinformatics Institute Arrayexpress for gene expression data [7] and NCBI GenBank [8]).

In the next section, we describe the BKR project corresponding to the post-publication stage.

### The Biomedical Knowledge Repository project (*Post-publication*)

**Figure 3:**
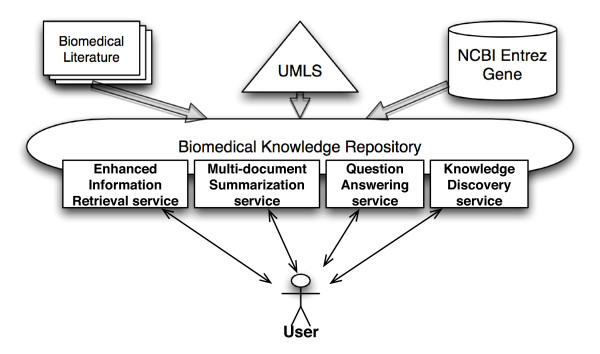
**Overview of the Biomedical Knowledge Repository (BKR) project**. The BKR project integrates data from three primary sources, namely scientific literature in PubMed, structured data in Entrez Gene, and the Unified Medical Language System (UMLS) terminological knowledge source, in RDF format. BKR offers four services using the integrated data namely, (a) enhanced information retrieval (using named relationship as search criteria), (b) multi-document summarization (using the confidence value associated with each assertion for ranking results), (c) question answering (allowing restriction of results to reputable journals or curated databases), and (d) knowledge discovery service using reasoning rules.

In contrast to the *T.cruzi *SPSE project, the Biomedical Knowledge Repository (BKR) project at the U.S. National Library of Medicine is creating a comprehensive repository of integrated biomedical data from a variety of published data sources such as biomedical literature (textbooks and journal articles), structured data bases (for example the NCBI Entrez system), and terminological knowledge sources (for example, the Unified Medical Language System (UMLS)) [9]. Similar to many biomedical data repositories [10], BKR uses W3C recommended Resource Description Framework (RDF) format [11] to represent the extracted and integrated information (Figure 3).

In addition to data, BKR project also includes provenance describing the source of an extracted RDF triple, temporal information (publication date for an article), version of a data repository, and confidence value associated with the extracted information (indicated by a text mining tool). For example, the provenance of the RDF statement "lipoprotein→affects→inflammatory_cells", the source article with PubMed identifier PMID: 17209178, is also stored in the BKR project (courier new font is used to represent RDF and OWL statements). The provenance information is used to support the services offered by BKR namely, (a) *Enhanced information retrieval service *that allows search based on named relationship between two terms, (b) *Multi-document summarization*, (c) *Question answering*, and (d) *Knowledge discovery service*.

**Figure 4:**
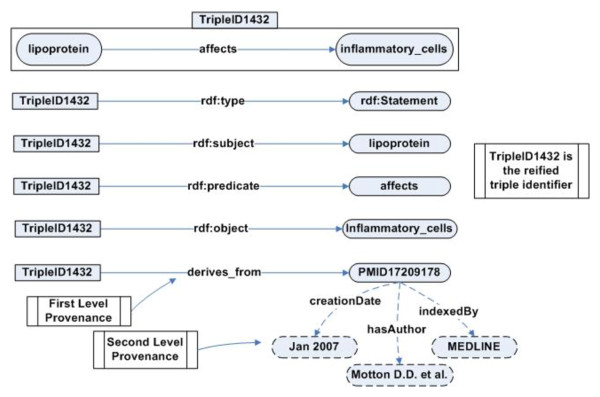
**Total number of RDF triples generated using the RDF reification vocabulary**. The reification of a single RDF triple leads to the creation of four extra RDF triples that do not model any provenance- related information but are merely artifacts of the RDF reification syntax.

The RDF reification vocabulary is often used to represent provenance information in Semantic Web applications. A variety of practical and theoretical issues have been identified in use of the RDF reification vocabulary [12,13], including a disproportionate increase in total size of the RDF document without a corresponding increment in the information content of the RDF document. Figure 4 illustrates this issue, where the reification of a single RDF triple leads to the creation of four extra RDF triples. The extra triples do not model any provenance-related information, but are merely artifacts of the RDF syntax. This adversely affects the scalability of large projects, such as BKR, which track the provenance of hundreds of millions of RDF triples.

### Challenges to provenance management in translational research

Broadly, the challenges to provenance management, in both the pre and post-publication stages, can be divided into four categories:

(a) **Collecting provenance information **in high throughput environments that is also adequate to support complex queries,

(b) **Representing the provenance information **using a model that supports interoperability across projects, is expressive enough to capture the complexities of a specific domain (*domain semantics*), and allows use of reasoning software for automated provenance analysis over large datasets,

(c) **Efficiently storing **and ensuring seamless **propagation **of provenance as the data is transferred across the translational research lifecycle,

(d) A dedicated **query infrastructure **that allows composition of provenance queries with minimal user effort, addresses the requirements specific to provenance queries (e.g., support for transitive closure), and a **highly scalable implementation **to support complex user queries over large volumes of data.

This paper extends our previous work [14,15] that separately addressed some aspects of provenance management in the pre and post-publications phases. In this paper, our contributions go beyond the previous work and can be summarized as follows.

- We introduce a unified provenance management framework called semantic provenance framework based on the Provenir upper-level provenance ontology for use in both the pre- and post-publication phases of translational research.

- We introduce a dedicated ontology-driven provenance collection infrastructure called Ontology-based Annotation Tool (OntoANT) that makes it easier for biomedical researchers to create and maintain web forms for use with bench experiments.

- We illustrate the advantage of storing provenance metadata and data as a single RDF graph with significant impact on propagation of provenance.

- We present the architectural details of a provenance query engine that can be deployed over multiple RDF databases and supports a set of dedicated provenance query operators.

In the next section, we describe SPF based on the notion of *semantic provenance *to address the provenance management challenges.

## Methods

In contrast to traditional database and workflow provenance, *semantic provenance *incorporates domain-specific terminology represented using a logic-based formal model, which facilitates domain scientists to intuitively query provenance and also automated processing of provenance metadata [16]. The *semantic provenance framework *(SPF) uses the Provenir upper-level provenance ontology as the *core *formal model coupled with Semantic Web technologies, including RDF [11], the Web Ontology Language (OWL) [17], and the SPARQL query language [18] for implementing provenance systems.

**Figure 5:**
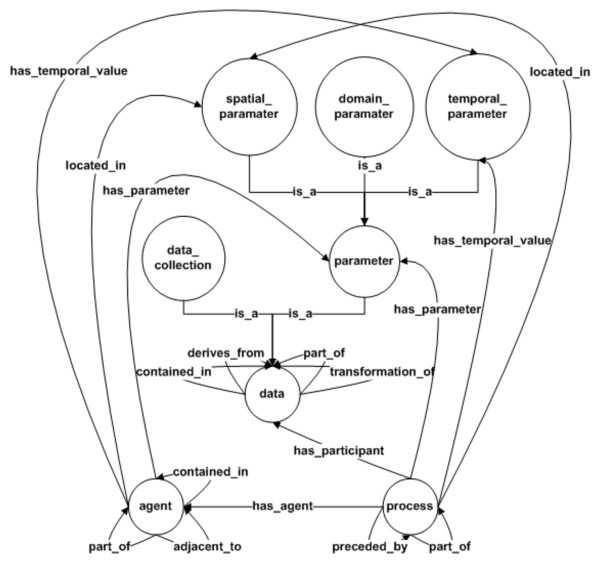
**Schema of the Provenir upper-level provenance ontology**. The Provenir ontology consists of three top-level classes namely, data, process, agent, and five sub-classes representing spatio-temporal and domain-specific parameters, and the data entities. These 8 classes are linked to each other using 11 named relationships that capture the formal semantics of the connections between the classes.

The approach used for provenance representation has a significant impact on the storage, propagation, and querying phases of the provenance life cycle. In [14], we had introduced the Provenir ontology as a reference model for provenance representation, which models a minimum set of provenance terms and relationships that are common across multiple translational research domains. The Provenir ontology extends primitive philosophical ontology terms of "continuant" and "occurrent" [19] along with ten fundamental relationships defined in the Relation ontology [20]. The Provenir ontology is composed of three top-level classes, namely data, process, and agent, which are fundamental to provenance modeling (Figure 5). The data class is further specialized into two classes namely, data_collection and parameter. The data_collection class represents entities that participate in an experiment or analysis process, while the parameter class, specialized into three classes along the spatial, temporal, and thematic (domain-specific) dimensions, models parameter values of a process. The Provenir ontology classes are linked by ten relationships adapted from the Relation ontology [20], which allows Provenir to capture and explicitly represent the semantics of the connections between terms that can be used by automated reasoning tools [21] for consistent interpretation of provenance.

The Provenir ontology is domain-upper ontology that can be extended, using the standard rdfs: subClassOf and rdfs: subPropertyOf[22] properties, for creating new domain-specific provenance ontologies. This approach of creating a suite of domain-specific ontologies by extending an upper-level ontology (instead of an unwieldy monolithic provenance ontology) facilitates provenance interoperability by ensuring consistent modeling and uniform use of terms [23] and is a scalable solution. This approach is also consistent with existing ontology engineering practices based on the Suggested Upper Merged Ontology (SUMO) [24], Basic Formal Ontology (BFO) [19], and the Descriptive Ontology for Linguistic and Cognitive Engineering (DOLCE) [25]). The Provenir ontology is modeled using the description logic profile of OWL (OWL-DL) [17].

In the following sections, we discuss the use of the Provenir ontology to implement the SPF for managing the four stages of the provenance metadata in the two translational research exemplar projects.

### Provenance Collection

The first phase of the provenance life cycle begins with the collection of provenance information as data is generated or modified in a project. The challenges in this phase include, (a) minimizing the disruption to existing research environment, (b) automating the collection procedure to scale with high-throughput data generation protocols while minimizing the workload for researchers, and (c) creating a flexible infrastructure that can be easily modified in response to changing user requirements. In the following sections, we describe the provenance collection infrastructure created for the *T.cruzi *SPSE and the BKR projects.

### Collecting provenance in the *T.cruzi *SPSE

**Figure 6:**
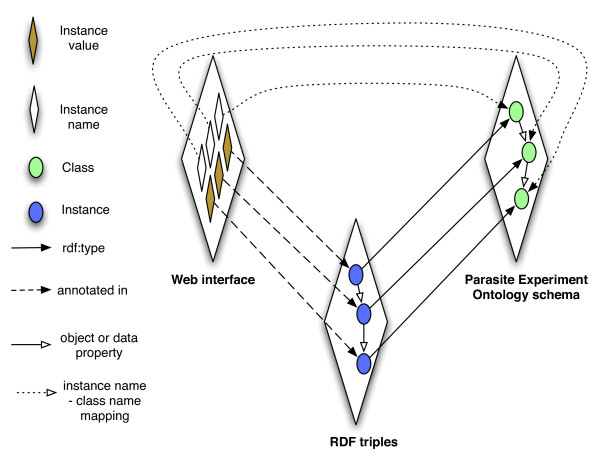
**Overview of the process to collect provenance and generate RDF triples from web forms in conjunction with the Parasite Experiment ontology**. The OntoANT tool involves interfacing of the domain-specific provenance ontology (PEO in case of the *T.cruzi *SPSE), the web forms used to collect the provenance information, and the structure of the RDF triples generated from the data captures in the web forms. This ensures consistency of the web form data with the domain-specific provenance ontology.

We used a two-phase approach to implement the provenance collection infrastructure in the *T.cruzi *SPSE. In the first stage, existing data stored in a RDB store was converted to RDF using the Parasite Experiment ontology (PEO), which represents the domain-specific provenance information, as reference. The D2RQ RDB to RDF tool [26] was used to convert the existing data in RDB to RDF by defining mapping between the data value in a RDB table column to a concept in the ontology (Figure 6). This batch conversion of data from the relational data to RDF was a temporary solution, while we created an integrated infrastructure to collect and directly store provenance information in RDF. To implement this infrastructure, we defined a novel ontology-driven web form generation tool called Ontology-based Annotation Tool (OntoANT). OntoANT allows domain scientists to:

1. Dynamically generate web forms for use in research projects to capture provenance information,

2. Allow automatic conversion of the data captured in the web forms to RDF, and

3. Use the built-in automatic validation of the web forms to ensure data quality and consistency with respect to the reference domain-specific provenance ontology (e.g. PEO)

**Figure 7:**
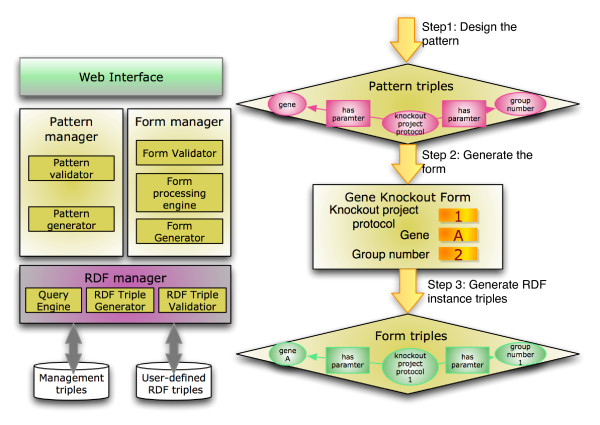
**The Ontology -based Annotation Tool (OntoANT) architecture and process flow to create a web form**. The OntoANT tool consists of three primary components, namely (a) the Pattern manager, (b) Form manager, and (c) the RDF manager. The Pattern manager and the Form manager have intuitive Web interface to allow easy creation and modification of ontology-based web forms used to collect provenance information. The RDF manager allows programmatic access to the functionalities of the Form and the Pattern manager components for developers.

**Figure 8:**
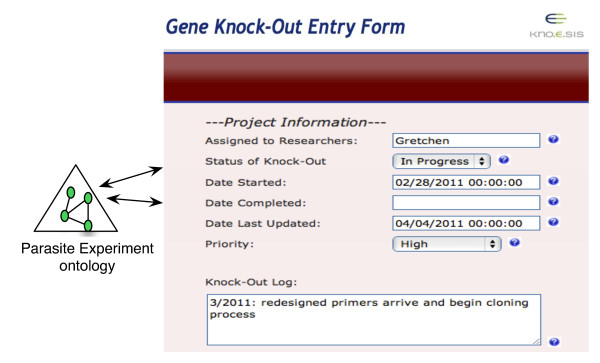
**Screenshot of an automatically generated web form to capture provenance information in the *T.cruzi *SPSE**. A web form created using the OntoANT tool to capture project-specific provenance information, including the name of the researcher, the status of an experiment process, and the start date of the experiment. The fields in the web form are mapped to the PEO classes and the values collected through the web forms are stored as RDF instance values of PEO.

OntoANT has three components (Figure 7), (1) a Pattern Manager, (2) a Form Manager, and (3) RDF Manager along with an intuitive web interface to allow domain users to easily manage the provenance collection infrastructure. The Pattern Manager in turn has two components, namely (a) a Pattern Generator, which is a visual interface to assist users in defining a "provenance pattern" to capture the relevant provenance information and is composed of provenance ontology classes and properties. For example, to create the "gene knockout entry form" (Figure 8), the user selects gene_knockout_process, researcher, and priority classes from PEO to compose the provenance pattern. The provenance pattern is used as reference by OntoANT to generate RDF triples from the data captured in the web form (Figure 7). The Pattern Validator, which is the second component of the Pattern Manager, validates the consistency of the provenance pattern with respect to the provenance ontology schema using the Pellet reasoning tool [21].

Once a valid provenance pattern is created, the Form Manager is invoked to automatically generate and deploy the web form. The Form Generator component of the Form Manager automatically generates the web form as a set of paired entities, namely, a field name and the corresponding text box (or a drop down list in case of a "nominal" class). Each field name in a web form corresponds to the provenance ontology class, while the value in the text box represents the instance of the ontology class (Figure 8). For example, the field "Priority" (Figure 8) corresponds to the priority class in PEO and the values in the drop-down menu (*High*, *Medium*, and *Low*) correspond to the instance values. The Form Processing Engine component of the Form Manager allows users to modify the automatically generated form. The third component of the Form Manager is the Form Validator, which ensures that the data values entered in the web forms are consistent with the provenance ontology. For example, the Form Validator validates that for a user input value for a web form field is consistent with the ontology class definition or with the property range or domain constraints.

The RDF Manager component of OntoANT defines a set of Application Programming Interfaces (API) that can be used by other OntoANT components to access, construct queries, and generate as well as validate RDF triples. OntoANT is currently being used in the *T.cruzi *SPSE project to deploy web forms (OntoANT is accessible at: http://knoesis.wright.edu/OntoANT/design.jsp).

### Provenance extraction in BKR

BKR collects the provenance information at two levels. At the first level the provenance information associated with an RDF triple is collected, such as the source of the triple (journal article, data repository), the date of the original publication, and the author list for the source article. At the second level, BKR records the provenance information associated with the extraction process, for example the confidence value associated with the extraction technique (in case of text processing tools). The provenance collection process in BKR is integrated with the RDF generation process, which is described in the next section on provenance representation. Provenance representation is a central issue in provenance management and has direct impact on the storage, querying, and analysis of provenance information in translational research.

### Provenance Representation

Earlier, we had described the Provenir ontology that forms the core model of the SPF. In this section, we demonstrate that though the requirements for provenance representation in the pre-publication phase differ from the post-publication phase, the Provenir ontology can be extended to model provenance in both the *T.cruzi *SPSE (pre-publication) and BKR (post-publication) projects.

### Parasite Experiment ontology: Modeling provenance in the *T.cruzi *SPSE project

**Figure 9:**
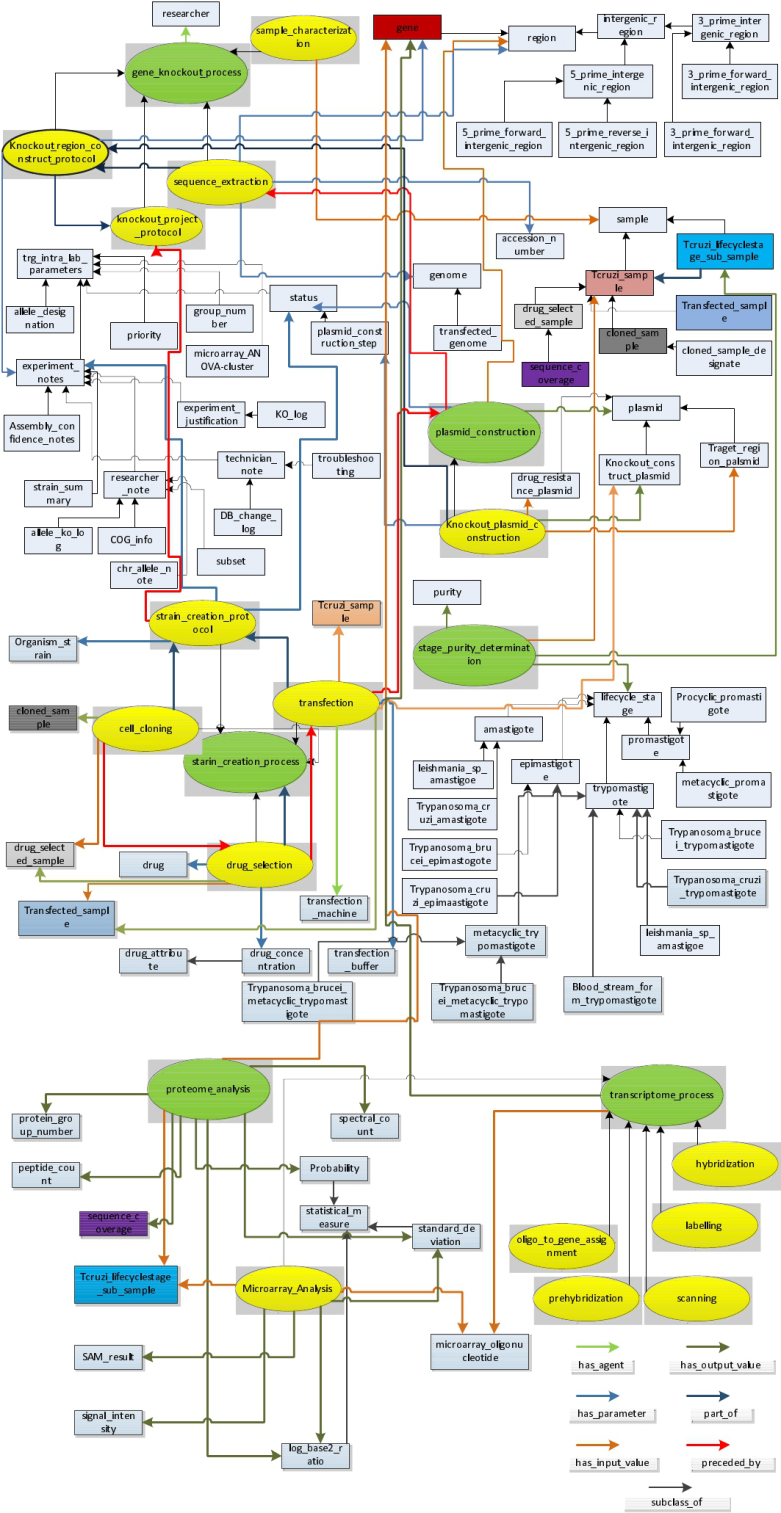
**Schema of the current version of the Parasite Experiment ontology**. The PEO schema models provenance information associated with four experiment protocols used in the *T.cruzi *SPSE, namely Gene Knockout, Strain Creation, Microarray, and Mass Spectrometry (MS) based proteomics. PEO not only extend the Provenir ontology classes, but also properties to define an extensive set of named relationships that are specific to the parasite domain.

In the pre-publication phase of translational research, the provenance information often describes the generation, curation, and processing of scientific data. In the *T.cruzi *SPSE project, the Parasite Experiment ontology (PEO) was created to model the experiment process used to generate data, the description of the raw material used, instruments, and parameter values that influence the generation or processing of data. In contrast to workflow provenance approaches that often model the "system-level" view of scientific processes [16], PEO incorporates domain-specific detail that allows us to comprehensively capture the context of an experiment and also allows researchers to use the domain-specific terminology to access and query the datasets. PEO initially modeled the experiment protocols used in reverse genetics (Gene Knockout and Strain Creation) as was reported in our previous work [14]. Currently, PEO has been extended to model Microarray as well as Mass Spectrometry (MS) based proteomics protocols also (Figure 9) and currently has 144 classes with 40 properties with a DL expressivity of ALCHQ(D).

PEO models the experiment protocols by specializing the Provenir ontology classes and properties. In addition, PEO re-uses classes and relationships from existing biomedical ontologies, including the Sequence ontology [27], the National Cancer Institute (NCI) thesaurus, Gene ontology [28], the W3C OWL Time ontology [29], and the Ontology for Parasite Lifecycle (OPL) [14]. This facilitates interoperability of data modeled using PEO with data that conform to other existing ontologies [30]. For example, we imported and seamlessly integrated functional gene annotations, using GO terms, from the TritrypDB [31] and KEGG with existing internal experiment data for a specific list of genes found in *T.cruzi *and related parasites. Hence, PEO creates a unified schema for both the domain-specific provenance information and data that can be extended (often re-using existing ontology classes) to adapt to evolving needs of bench scientists in the *T.cruzi *SPSE project.

### Provenance Context: Representing provenance in the BKR project

**Figure 10:**
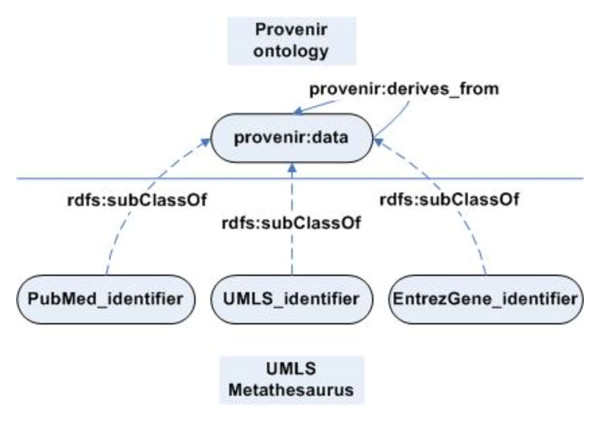
**The provenance context for the BKR project**. The PaCE approach apriori defines the provenance context for a project that is used to create RDF triples that incorporate the specified provenance context. The provenance context of the BKR project consists of the three data sources, PubMed, UMLS Metathesaurus, and Entrez Gene, from which information is extracted and integrated in RDF format.

In contrast to the *T.cruzi *SPSE project, the representation of provenance in the BKR project was more challenging. As we discussed earlier, the traditional RDF reification approach has many limitations that makes it difficult for translational research projects such as BKR to use it for provenance tracking. To address the limitations of the RDF reification approach, we defined a new approach based on the Provenir ontology and context theory called Provenance Context Entity (PaCE) [15]. The premise for the PaCE approach is that the provenance associated with RDF triples provides the contextual information necessary to correctly interpret RDF statements. A "provenance context" is defined for a specific application, such as BKR, by either using terms of the Provenir ontology or terms defined in a domain-specific provenance ontology that extends the Provenir ontology. In the BKR project (Figure 10), the provenance context consists of the Unified Medical Language System (UMLS) Semantic Network (SN) [32] terms, Entrez Gene and PubMed identifiers. The terms of the BKR provenance context are defined as subclass of provenir: data class using the rdfs: subClassOf property.

**Figure 11:**
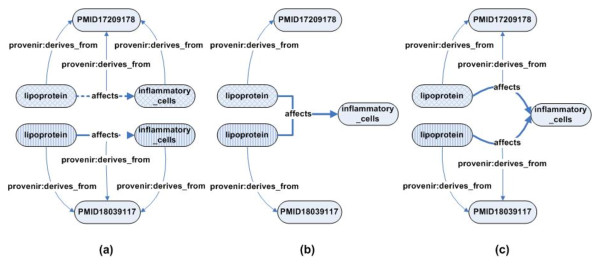
**The three PaCE implementations with different levels of granularity**. The PaCE approach allows applications to choose a desired level of granularity for representing provenance of the components of a RDF triple. The three approaches vary from (a) an exhaustive approach that explicitly models provenance of the S, P, and O componets of a triple, to (b) a minimalist approach that tracks the provenance of only the S of a triple, and finally (c) an intermediate approach that tracks the provenance of the P (in addition to the S) of a triple. There are advatanges and disadvantages associated with each of the three approaches that need to be considered by an application.

Once a provenance context has been defined for an application, the contextualized RDF triples can be generated from the information extracted from the original data sources. The PaCE approach allows an application to decide the appropriate level of granularity with three possible implementation approaches. The first implementation (Figure 11) is an exhaustive approach and explicitly links the S, P, and O to the source journal article. The second implementation is a minimalist approach that links only the S of a RDF triple to the source article. The third implementation takes an intermediate approach that creates two additional provenance-specific triples but requires the application to assume that the source of the O is the same as the S, and P. It is important to note that none of the three variants of the PaCE approach requires the use of RDF reification vocabulary or the use of blank nodes.

A practical challenge for implementing the PaCE approach in the BKR is to formulate an appropriate provenance context-based Uniform Resource Identifier (URI_p_) scheme that also conforms to best practices of creating URIs for the Semantic Web, including support for use of HTTP protocol [33]. The design principle of URI_p _is to incorporate a "provenance context string" as the identifying reference of an entity and is a variation of the "reference by description" approach that uses a set of description to identify an entity [33]. The syntax for URI_p _consists of the <base URI>, the <provenance context string>, and the <entity name>. For example, the URI_p _for the entity lipoprotein is http://mor.nlm.nih.gov/bkr/PUBMED_17209178/lipoprotein where the PUBMED_17209178 provenance context string identifies the source of a specific instance of lipoprotein.

This approach to create URIs for RDF entities also enables BKR (and other Semantic Web applications using the PaCE approach) to group together entities with the same provenance context. For example,

http://mor.nlm.nih.gov/bkr/PUBMED_17209178/lipoprotein

http://mor.nlm.nih.gov/bkr/PUBMED_17209178/affects

http://mor.nlm.nih.gov/bkr/PUBMED_17209178/inflammatory_cells

are entities extracted from the same journal article. The multiple contextualized URIs representing a common type of entity, for example "lipoprotein", can be asserted to be instances of a common ontology class by using the rdf: type property. In the next section, we address the issues in provenance storage and propagation stage of the provenance lifecycle.

### Provenance Storage and Propagation

The current high-throughput data generation techniques, including gene sequencing and DNA microarray, have created very large datasets in biomedical applications [7,8]. Though the capture and storage of provenance associated with the above datasets leads to an exponential increase in the total size of the datasets [34], provenance plays an important role in optimizing the access and query of the datasets [35,14]. There are two approaches to store provenance, namely (a) provenance is stored together with the dataset, and (b) provenance is stored separately from the data (and combined on demand).

The SPF uses the first approach by storing both the data and provenance together in a single RDF graph. The primary motivation for selecting the first approach is to allow applications to flexibly categorize an information entity as either data or provenance metadata according to evolving user requirements. For example, the temperature of a gene knockout experiment (in the *T.cruzi *SPSE project) is provenance information, which can be used to query for results generated using similar temperature conditions. In contrast, the body temperature of a patient in clinical research scenario is a data value and not provenance information. Hence, this application-driven distinction between provenance metadata and data is a critical motivation for storing provenance and data together in the SPF. In addition, storing provenance together with the data makes it easier for application to also ensure that updates to data are seamlessly applied to the associated provenance. Ensuring synchronization between the data and separately stored provenance is challenging especially in a high-throughput data generation scenarios and the provenance information may become inconsistent with the data.

An essential requirement for provenance storage is ensuring the propagation of provenance as the data traverses the translational research life cycle, for example the provenance of gene expression profiling experiment results is used in a downstream application such as biological pathway research. The integrated approach for provenance storage allows seamless propagation of provenance information with the data. In contrast, it is often difficult to transfer provenance separately from the data across projects, institutions, or applications. Further, many applications often query the provenance metadata to identify relevant datasets to be imported and analyzed further, for example identifying a relevant patient cohort for clinical research requires identifying qualifying health care providers, the geographical location of the patients, and related provenance information. Hence, if the provenance associated with a patient health record is stored separately and cannot be easily propagated and accessed by the clinical researcher then it adversely affects translational research projects.

Though storing provenance and data together has many advantages, one of the challenges that needs to be addressed is the large size of the resulting datasets. Cloud-based storage solutions, such as Simple Storage Service (S3) from Amazon and Azure blob from Microsoft have been proposed to effectively address these issues [35].

### Provenance storage in the *T.cruzi *SPSE

The *T.cruzi *SPSE project currently stores more than 700,000 RDF triples corresponding to the data and the associated provenance information for four experiment protocols, namely Proteome, Microarray, Gene Knockout, and Strain creation (Table 1). The experiment data and the associated provenance information are stored in a single RDF graph. This allows easy propagation of provenance along with the original experiment data. The RDF triples are stored in an Oracle10g (Release 10.2.0.3.0) RDF datastore. Table 1 illustrates that a very large percentage of the total data, between 87% (for Strain Creation experiment protocol) to 98% (for Gene Knockout experiment protocol), is provenance information. The use of provenance information to query and access specific datasets is discussed later in the Provenance Query and Analysis section.

**Table 1:** Details of the RDF instance base in the *T.cruzi *SPE project

Experiment Protocol	Number of Experiment Runs	Total RDF Triples	Provenance-specific RDF Triples (*% of total triples*)
1. Proteome analysis	3764	283,883	259,903 (*91%*)

2. Microarray	14,100	476,105	466,153 (*97%*)

3. Gene Knockout	151	14,632	14,371 (*98%*)

4. Strain Creation	82	3,111	2,747 (*87%*)

### Provenance storage in the BKR project

In the BKR project, the initial data (without the provenance information) consisted of 23.4 million RDF triples. The initial data was augmented with the provenance information and stored using the three PaCE approaches discussed in the previous section (Table 2), namely:

**Table 2:** Number of provenance-aware RDF triples generated using the PaCE and RDF reification vocabulary

	PaCE Minimal	PaCE Intermediate	PaCE Exhaustive	RDF Reification vocabulary
Total Number of RDF triples	71,765,914	94,766,314	113,143,327	175,592,122

Provenance-specific RDF triples	48,332,257	71,332,657	89,709,670	152,158,465

a) Exhaustive approach (E_PaCE): Capturing the provenance of the S, P, and O elements of the RDF triple increased the total size of the BKR dataset to 113.1 million RDF triples

b) Minimal approach (M_PaCE): 48.3 million additional RDF triples (total 71.6 million RDF triples) were created using this approach

c) Intermediate approach (I_PaCE): A total of 94.7 million RDF triples were created using the I_PaCE approach

Table 2 also clearly illustrates the decrease in the number of provenance-specific RDF triples as compared to the RDF reification vocabulary approach. The open source Virtuoso RDF store version 06.00.3123 was used to store the BKR datasets. Similar to the *T.cruzi *SPSE project, the provenance metadata associated with the BKR data is seamlessly propagated along with the data since both are represented in a single RDF graph.

### Provenance Query and Analysis

The provenance literature discusses a variety of queries that are often executed using generic or project-specific query mechanisms that are difficult to re-use. Provenance queries in workflow systems focus on execution of computational process and their input/output values. Provenance queries in relational databases trace the history of a tuple or data entity [36]. In contrast, scientists formulate provenance queries using domain-specific terminology and follow the course of an experiment protocol [16,37]. In addition, provenance queries over scientific data often exhibit "high expression complexity" [38] reflecting the real world complexity of the scientific domain [14].

The composition of provenance queries using SQL or SPARQL query languages is not intuitive for translational research scientists. Hence, we have defined specialized "query operators" for use by domain scientists, which use the specified input value (Table 3) to automatically compose and execute complex provenance queries:

**Table 3:** Provenance query operator input and output value

Provenance Query Operator	Input Value	Output Value	Implementation Language
1. *provenance ( )*	Data entity (instance of Provenir data_collection class)	Provenance of data entity	SPARQL

2. *provenance* *_context ( )*	Provenance of data entity (instances of Provenir data, agent, and process classes)	Data entity(s) (satisfying the provenance constraints)	SPARQL

3. *provenance* *_compare ( )*	Provenance of two data entities (RDF files)	True (if provenance of two data entities are equivalent), otherwise False	SPARQL

4. *provenance* *_merge ( )*	Two sets of provenance information (RDF files)	Merged provenance information	SPARQL

a) **provenance ( ) **query operator - to retrieve provenance information for a given dataset,

b) **provenance_context ( ) **query operator **- **to retrieve datasets that satisfy constraints on provenance information,

c) **provenance_compare ( ) **query operator - given two datasets, this query operator determines if they were generated under equivalent conditions by comparing the associated provenance information, and

d) **provenance_merge ( ) **query operator - to merge provenance information from different stages of an experiment protocol. In the *T.cruzi *SPSE project, provenance information from two consecutive phases, namely gene knockout and strain creation phases, can be merged using this query operator.

The query operators are defined in terms of a "search pattern template" composed of Provenir ontology classes and properties (the query operators are defined using formal notation in [16]). The query operators use the standard RDFS entailment rules [12] to expand the query pattern and can be executed against the instance base of any (Provenir-ontology based) domain-specific provenance ontology. The formal definition of these query operators is described in [16]. In addition, the query operators can be extended to create new query operators and can be implemented in either SQL or SPARQL.

## Results

The SPF was implemented as a scalable provenance query engine that can be deployed over any RDF database that supports standard RDFS entailment rules [12].

### Provenance Query Engine

**Figure 12:**
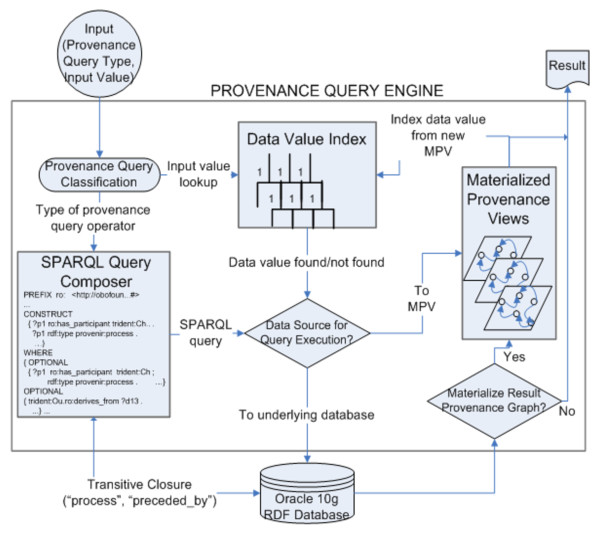
**Architecture of the provenance query engine**. The provenance query engine implements the four provenance query operators defined as part of the SPF and can be deployed over any RDF database that supports the standard RDFS entailment rules. The query engine consists of three components, to compose a SPARQL query pattern corresponding a query operator, a module to compute the transitive closure over the <process, preceded_by> class-property pair, and an optimization module that used the schema of the provenance ontology to materialize a RDF sub-graph to answer multiple provenance queries.

The provenance query engine consists of three functional components (Figure 12):

#### 1. **A Query Composer**

The query composer maps the provenance query operators to SPARQL syntax according to semantics of the query operators.

#### 2. **A Function to Compute Transitive Closure over RDF**

SPARQL query language does not support transitive closure for an RDF <*node*, *edge*> combination. Hence, we have implemented a function to efficiently compute transitive closure using the SPARQL ASK function. The output of this function together with the output of the query composer is used to compose the complete query pattern.

#### 3. **Query Optimizer using Materialized Provenance Views**

Using a new class of materialized views based on the Provenir ontology schema called Materialized Provenance Views (MPV) a query optimizer has been implemented that enables the query engine to scale with very large RDF data sets.

The query operators are implemented taking into account the distinct characteristics of provenance queries as well as existing provenance systems. For example, provenance information represents the complete history of an entity and is defined by the exhaustive set of dependencies among data, process, and agent. However, in real world scenarios the provenance information available can be incomplete due to application-specific or cost-based limitations. Hence, a straightforward mapping of provenance query operators to SPARQL as a Basic Graph Pattern (BGP) is not desirable, since the BGP-based query expression pattern may not return a result in the presence of incomplete provenance information [18]. Hence, the OPTIONAL function in SPARQL can be used to specify query expression patterns that can succeed with partial instantiation, yielding maximal "best match" result graph. Another challenge in implementation of the query engine was that unlike many graph database query languages such as Lorel or GraphLog, [39], SPARQL does not provide an explicit function for transitive closure to answer reachability queries (http://www.w3.org/2001/sw/DataAccess/issues#accessingCollections). Reachability queries involving computation of transitive closure is an important characteristic of provenance queries to retrieve the history of an entity beginning with its creation. In case of the provenance query engine, the query composer computes the transitive closure over the <process, preceded_by> combination to retrieve all individuals of the process class linked to the input value by the preceded_by property.

### Transitive Closure Module

**Figure 13:**
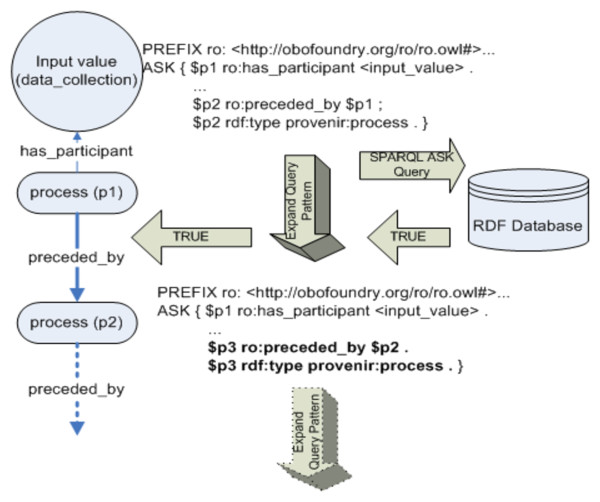
**RDF transitive closure using SPARQL ASK function**. Computing transitive closure is a distinct feature of provenance queries, as the history of an entity is traced to its origin. Since the provenance query operators are implemented in SPARQL and SPARQL does not feature in-built support for computing transitive closure, the provenance query engine includes a module that uses the SPARQL ASK function to efficiently compute transitive closure over the <process, preceded_by> class-property pair.

We had two options in implementing the transitive closure function, namely a function that is tightly coupled to a specific RDF database or a generic function. We chose a generic implementation using the SPARQL ASK function that allows the provenance query engine to be used over multiple RDF stores. The SPARQL ASK function allows "application to test whether or not a query pattern has a solution," [18] without returning a result set or graph. The transitive closure function starts with the process instance (p1) linked to the input value and then recursively expands the SPARQL query expression using the ASK function till a *false *value is returned, thereby terminating the function (Figure 13). The SPARQL ASK function, in contrast to the SELECT and CONSTRUCT functions, does not bind the results of the query to variables in the query pattern. Hence, it is a low-overhead function for computing transitive closure [16].

The evaluation of the provenance query engine followed the standard approach in database systems [40] and was performed for both "*expression complexity*" - SPARQL query patterns with varying levels of complexity, and "*data complexity*" - varying sizes of RDF datasets. The SPARQL query complexity was measured using the total number of variables, triples, use of OPTIONAL function, and levels of nesting in the query pattern [41]. The most complex query pattern had 73 variables, 206 triples, and 7 levels of nesting using the OPTIONAL function. Further, to evaluate the *data complexity*, five different sized datasets were used ranging from 32,000 RDF triples to 308 million RDF triples. We found that a straightforward implementation of the query engine was not able to scale with both increasing expression and data complexity [16]. Hence, the provenance query engine uses a novel materialization strategy based on the Provenir ontology schema, called materialized provenance views (MPV) [16]. The use of MPV improved the performance of the query engine by an average of 99.93% for increasingly complex SPARQL query patterns and by an average of 98.95% for increasingly large RDF datasets, thereby validating the scalability of the query engine. We now describe a few example provenance queries in context of the *T.cruzi *SPSE project that leverage the SPF query infrastructure.

### Provenance queries in the *T.cruzi *SPSE

In the *T.cruzi *SPSE project, provenance queries broadly address two types of issues:

1. Retrieving the history of experiment results to ensure quality and reproducibility of data. In addition, the provenance information is used to describe the experiment conditions of results published in literature

2. Keeping track of experiment resources during an ongoing project or auditing of resources used in a completed project. This helps project managers to monitor status of projects and ensure optimal use of lab resources

We consider the following two example provenance queries representing the above two categories of usage:

**Query 1**: *Find the drug and its concentration that was used during drug selection process to create "cloned_sample66."*

**Query 2**: *What is the status of knockout plasmid construction step to create pTrex? *Query 1 illustrates the retrieval of provenance information associated with a cloned sample, where the type of drug and concentration of the drug are important for researchers to understand the characteristics of the cloned sample. Similarly, Query 2 describes a provenance query used for project management, where the lead researcher or project manager can keep track of the project status. Both these example queries are answered using the *provenance () *query operator, which takes as input "*cloned_sample66*" and "*pTrex*" as input values respectively.

As described earlier, the *provenance () *query operator, implemented in the provenance query engine, automatically generates a SPARQL query pattern using the PEO schema as reference. This query pattern is executed against the *T.cruzi *SPSE RDF instance base and the retrieved results are represented as a RDF graph (which can be used by any Semantic Web visualization tool, for example Exhibit [42] or in the Cuebee query interface [43]). Similar to our earlier work [14], the results of the above queries were manually validated by domain researchers in the Tarleton research group. In the next section, we describe provenance queries used in the BKR project.

### Provenance query in the BKR Project

The provenance queries in the BKR project are used for identifying the source of an extracted RDF triple, retrieving temporal information (for example, the date of publication of a source article), version information for a database, and the confidence value associated with a triple (indicated by a text mining tool). The provenance information is essential in the BKR project to ensure the quality of data and associate trust value with the RDF triple. We discuss the following two example provenance queries used in the BKR project:

**Query 1**: *Find all documents asserting the triple "IL-13 **→ **inhibits → COX-2"*

**Query 2**: *Find all triples of the form "IL-13 **→ **inhibits *→ *gene" where value of gene is not known apriori*. *The results are filtered based on a set of provenance constraints such that results are only from (a) journals with impact factor > 5, (b) journal published after the year 2007, (c) RDF triples with confidence value > 8*.

Query 1 is used by the enhanced information retrieval service in the BKR project, which supports user query based on not only keyword or concepts, but also relations [3]. Hence, results from Query 1 are used to create a basic index, similar to traditional search engines, listing all documents from which a given biomedical assertion is extracted [3]. In contrast to Query 1, Query 2 is used by the Question Answering service of the BKR project to define provenance-based quality constraints to retrieve results from reputable journals that have been published recently and a high confidence value is associated with the extracted RDF triple. Both the provenance queries are expressed in SPARQL and executed against the BKR instance base. In our earlier work, we have discussed the improved performance of provenance queries using the PaCE approach in comparison to the RDF reification vocabulary [15].

In both the *T.cruzi *SPSE and BKR project, the SPF provides users with an easy to use, expressive, and scalable provenance query infrastructure that can scale with increasing size of data and complexity of the queries [16,43].

## Discussions

We first discuss related work in provenance representation in context of the Provenir ontology. Next, we discuss the work in database provenance and workflow provenance with respect to provenance query/analysis and compare it with the functionality of the provenance query operators defined in SPF.

### Provenance representation

Multiple provenance representation models have been proposed, with the Open Provenance Model (OPM) [44] and the proof markup language (PML) [45] being the two prominent projects. As part of the W3C Provenance Incubator Group, we have defined a lightweight mapping between the OPM and other provenance models including the Provenir ontology, which demonstrates that all three of them model similar classes, but only the Provenir ontology has a comprehensive set of named relationships linking the provenance classes [46]. Specifically, OPM (core specification) models only "causal relations" linking provenance entities [44], which makes it difficult for OPM to model partonomy, containment, and non-causal participatory provenance properties needed in many translational research applications. Provenance representations, in the context of relational databases, extend the relational data model with annotations [47], provenance and uncertainty [36], and semirings of polynomials [48]. Provenir ontology can be extended to model the provenance of tuple(s) in relational databases, which relies on mappings defined between description logic to relational algebra [49].

### Database provenance

Database provenance or data provenance, often termed as "fine-grained" provenance, has been extensively studied in the database community. Early work includes the use of annotations to associate "data source" and "intermediate source" with data (polygen model) in a federated database environment to resolve conflicts [50], and use of "attribution" for data extracted from Web pages [51]. More recent work has defined database provenance in terms of "Why provenance," "Where provenance," [52] and "How provenance" [48]. "Why provenance", introduced in [53], describes the reasons for the presence of a value in the result (of a query in a relational database context) and "Where provenance" describes the source location of a value [52]. A restricted view of the "Where provenance" identifies each piece of input data that contributes to a given element of the result set returned by each database query. We use the syntactic definition of "Why provenance" [52] that defines a "proof" for a data entity. The proof consists of a query, representing a set of constraints, over a data source with "witness" values that result in a particular data output. The semantics of the *provenance () *query operator closely relates to both "Where provenance" and "Why provenance" [52].

To address the limitation of "Why provenance" that includes "...set of all contributing input tuples" leading to ambiguous provenance, [48] introduced semiring-based "How provenance." The *provenance () *query operator over a "weighted" provenance model, which reflects the individual contribution of each component (for example process loops or repeated use of single source data), is comparable to "How provenance."

The Trio project [36] considers three aspects of lineage information of a given tuple, namely, how was a tuple in the database derived along with a time value (when) and the data sources used. A subset of queries in Trio, "lineage queries", discussed in [36], can be mapped both as *provenance () *and as *provenance_context () *query operators depending on the input value.

### Workflow provenance

The rapid adoption of scientific workflows to automate scientific processes has catalyzed a large body of work in recording provenance information for the generated results. Simmhan et al. [54] survey different approaches for collection, representation, and management of workflow provenance. Recent work has also recognized the need for inclusion of domain semantics in the form of domain-specific provenance metadata [16] along with workflow provenance [55]. The semantics of these projects can be mapped to the *provenance () *query operator.

**Figure 14:**
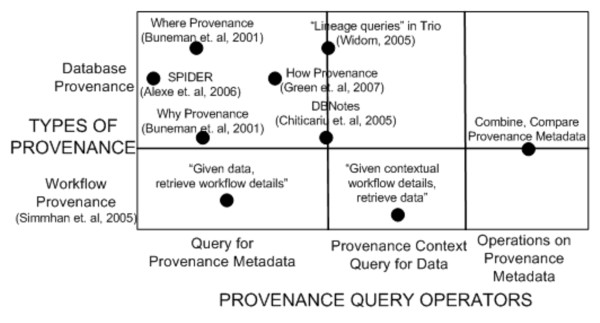
**Mapping provenance query operators with existing database and workflow provenance**. The figure maps the functionality of the provenance query operators defined in the SPF to existing provenance work in both the database community and the scientific workflow community.

Figure 14 describes the mapping of the SPF query operators to existing work in both database and workflow provenance.

## Discussions

In our previous work [14,15], we have separately addressed some of the issues in pre- and post-publications phases of translational research applications. Here we expand on the challenges in creating a unified framework for provenance management, with a focus on a dedicated infrastructure for effective provenance collection, a flexible provenance model, and a scalable query implementation that can be adopted across translational research projects.

*What does it take to build an effective provenance management system for translational research today? *It is clear from the work discussed in this paper that creation of a practical and usable provenance management system is not a trivial task. Though provenance represents critical information for research projects, the high threshold in terms of resources required deters widespread adoption of a systematic and comprehensive provenance infrastructure. In addition, the lack of provenance-specific standards makes it difficult for developers to implement interoperable provenance systems across projects, applications, and different phases of the translational research lifecycle. This current state of provenance systems forces researchers to create ad-hoc systems that cannot be re-used, extended, or adapted to changing project requirements.

Hence, we have deliberately aligned the implementation of the SPF components with existing W3C Semantic Web standards, including RDF, OWL, and SPARQL. Though, these standards are not tailored for the specific requirements of provenance systems, we demonstrated that they can be extended and adopted to address some of the challenges. For example, a component of the provenance query engine uses SPARQL ASK function to compute transitive closure over RDF graphs, since SPARQL does not have explicit support for computing transitive closure. Despite some advantages of using existing Semantic Web standards, provenance management in context of translational research is still in an early phase.

*How are things likely to improve in the future? *The W3C provenance incubator group (Provenance XG) [46] has collected an extensive set of use cases and requirements for effective provenance management. This work has led to the creation of the W3C Provenance Working Group, which has been mandated to define a language for exchanging provenance information across applications [46]. In addition, the working group will also define a mechanism for querying and accessing the provenance information along with a set of best practices that can be used to guide implementation of provenance systems [46]. We are members of the working group and we plan to make the SPF compatible with the standards that will be proposed by the working group.

## Conclusions

We described a unified framework based on the upper-level Provenir provenance ontology for managing provenance information during generation of data from bench experiments and their subsequent use (post-publication) by data mining and knowledge discovery applications. In the process, we identified that both the pre and post-publication phases of translational research have a common set of stages associated with the provenance metadata that can be managed by the SPF. Using two exemplar projects, corresponding to the two translational research phases, we described how the SPF could handle provenance collection, representation, storage/propagation, and query/analysis.

As part of our future work, we will implement a "lifting mechanism" between contexts to allow easier transformation of RDF triple between different PaCE-based applications. In addition, we aim to specialize the existing provenance query operators to interface with distributed SPARQL end-points, which have been proposed for provenance access and querying by the W3C Provenance Working Group.

## Authors' contributions

SSS defined the framework, created the ontologies, implemented query operators, query engine, and wrote this manuscript. VN designed and implemented the OntoANT tool and contributed to writing of the manuscript. OB implemented PaCE, executed the queries, and validated the results. PP and TM defined the *T.cruzi *SPSE queries, contributed to PEO, and validated the results. APS contributed to creation of the framework and development of the ontologies. All authors have read and approved the final manuscript.
